# Classification of daily activities using a wireless instrumented insole (WalkinSense) in a semi free-living setting

**DOI:** 10.1371/journal.pone.0357961

**Published:** 2026-09-16

**Authors:** Anne Backes, Melanie Eckelt, Jennifer Fayad, Valeria Serchi, Thomas Solignac, Tobias Meyer, Bernd Grimm, Caroline Mouton, Romain Seil, Laurent Malisoux

**Affiliations:** 1 Department of Precision Health, Luxembourg Institute of Health, Strassen, Luxembourg; 2 Luxembourg Institute of Research in Orthopedics, Sports Medicine and Science, Eich, Luxembourg; 3 IEE S.A., Bissen, Luxembourg; 4 Centre Hospitalier de Luxembourg, Eich, Luxembourg; The Hong Kong Polytechnic University, HONG KONG

## Abstract

**Background:**

Accurate monitoring of activities of daily living (ADLs) in real‑world environments is essential for preventive care and rehabilitation, yet it remains difficult to achieve outside controlled laboratory settings. Instrumented insoles provide a promising, unobtrusive solution for continuous monitoring. However, the use of multimodal systems integrating plantar pressure and inertial data in naturalistic conditions and larger cohorts is still limited. This study therefore aims to evaluate the accuracy of a wireless instrumented insole (WalkinSense) that fuses pressure and inertial sensor data to classify ADLs within a semi free-living setting.

**Methods:**

A total of 99 participants performed a broad set of indoor and outdoor activities. Frame-by-frame performance was compared to ground truth (direct observation) using overall accuracy, Cohen’s Kappa, precision, recall, F1-scores and a normalised confusion matrix. Agreement on total activity duration was assessed using mean absolute percentage error (MAPE) scores and Bland-Altman plots.

**Results:**

Activity classification showed almost perfect agreement (mean overall accuracy 0.87, mean Cohen’s Kappa 0.84). Excellent performance (F1 > 0.90) was achieved for sitting, walking with crutches and cycling, while standing, level and non-level walking showed good performance (F1 > 0.80). Most misclassifications occurred between level walking, hill walking and stairs. Duration-based analysis confirmed high accuracy for sitting, walking with crutches and cycling (MAPE ≤ 10%). Bland-Altman plots indicated overestimation of level walking and underestimation of hill and stair walking. Step count was highly accurate (MAPE < 5%), whereas stair count showed only reasonable accuracy (MAPE ≈ 26%).

**Conclusion:**

These findings demonstrate the system’s strong potential for real-world monitoring and classification of ADLs while also highlighting the need for improved detection of non-level walking activities.

## Introduction

Accurate monitoring of physical activity behaviour in daily life plays an important role in preventive healthcare, rehabilitation, and the management of chronic conditions [1–3]. Identifying and quantifying activities of daily living (ADLs), such as walking, standing, sitting or climbing stairs, may support the assessment of functional capacity, the monitoring of recovery and the evaluation of fall risk in both healthy and clinical populations. Traditional gait laboratories, while offering high measurement precision, are limited by artificial testing environments, reduced ecological validity and high operational costs. These constraints restrict their ability to capture free‑living behaviour or conduct regular, longitudinal assessments [4]. Consequently, there is growing interest in wearable sensor technologies that enable continuous and unobtrusive monitoring of ADLs in naturalistic settings.

Among these technologies, instrumented insoles have gained considerable attention because they can be worn in footwear and capture detailed plantar pressure distributions alongside gait-related parameters without impeding natural movement [5,6]. The WalkinSense system (WSS) is one such promising solution, integrating pressure sensors and inertial measurement units (IMUs) in a wireless insole. While the WSS has been validated for spatiotemporal gait assessment [7,8], its potential for activity classification, especially in rehabilitation-oriented applications, remains underexplored.

Recent work has demonstrated that smart insoles can classify ADLs using a broad range of computational approaches, including classical statistical classifiers such as multinomial logistic discrimination [2,9], machine learning (ML) models such as k-nearest neighbours (k-NN), support vector machines (SVM) and random forests [9–18], as well as deep-learning (DL) architectures including multi-layer perceptron, artificial neural networks or convolutional neural networks [1,9,15,16,19]. These models have been trained using pressure data alone or multimodal combinations of pressure and inertial signals. However, much of the existing evidence has been generated in relatively small and homogeneous participant samples and under controlled laboratory conditions that do not capture the variability, noise and behavioural diversity characteristic of real‑world environments. Moreover, even when studies included a free‑living component, validation was typically performed using secondary device‑based comparators of limited accuracy instead of gold‑standard direct observation and only a narrow subset of ADLs was assessed [2,20]. To date, no study has combined large‑scale, ecologically valid, multimodal (pressure-inertial) insole data with direct observation as the reference standard to evaluate activity‑classification performance under conditions that approximate everyday use in rehabilitation contexts.

These methodological constraints have important implications for model selection. Although the abovementioned ML and DL architectures can achieve high classification accuracy in controlled settings, their performance under real‑world conditions remains uncertain. Complex models trained on small, homogeneous datasets are prone to overfitting to a narrow distribution of movement patterns and may show reduced reliability when confronted with the noisy, imbalanced and behaviourally diverse characteristics of semi free‑living data [21]. Consequently, when real-world generalisability and large-scale data collection are prioritised, the motivation for employing highly complex architectures becomes less compelling. Under these circumstances, the key challenge shifts from maximising accuracy in a constrained setting to developing a classifier that is robust, transparent and generalisable across participants and environments. Classical models such as classification trees are well suited to this purpose. They are resilient to real‑world variability, provide interpretable decision rules that clinicians and researchers can inspect and validate, and offer computational efficiency suitable for potential on‑device implementation within wearable systems.

Accordingly, the present study aims to evaluate the accuracy of a wireless instrumented insole that fuses plantar pressure and inertial data to classify ADLs collected in a semi free-living environment, using a classification tree model and direct observation as the reference standard. This approach addresses key gaps in the literature regarding real-world generalisability, large‑scale validation and the performance of multimodal insole systems for clinically relevant activities.

## Methods

### Study population

A total of 104 healthy participants with no history of lower-limb surgery or orthopaedic conditions were recruited between February 15 and September 8, 2023, to voluntarily participate in the present study. The sample size was determined for the overall GAITORING project, which aimed to validate the WSS against gold-standard laboratory equipment [7]. Based on agreement analyses following Lu et al. [22], between 39 and 83 participants were required depending on the outcome measure; therefore, a target of 100 participants was set to account for potential data loss [7]. The present activity classification analysis was conducted on the same cohort using an additional semi free-living protocol. No separate sample size calculation was performed; however, the included sample provides a sufficiently large and diverse dataset to support robust evaluation of classification performance. This study was conducted in accordance with the ethical standards of the Declaration of Helsinki and was approved by the National Ethics Committee for Research (CNER, reference: 202212/04 Version 2.0). Prior to participation, all participants received a comprehensive explanation of the study protocol and provided written informed consent.

### Equipment

The WSS (IEE S.A., Bissen, Luxembourg) comprises a hardware setup that integrates both an IMU (ICM-20948) and pressure-sensitive insoles for data acquisition. The system’s detailed specifications have been described previously [7]. In brief, each insole is equipped with eight high-definition force-sensitive resistor (HD-FSR) sensors. The insoles are compact, lightweight and designed for seamless integration into various types of footwear, allowing participants to wear their own shoes. The WSS supports continuous 24-hour monitoring with an extended battery life of up to 10 days and operates at a sampling frequency of up to 200 Hz. The WSS comprises a digital environment allowing for the full control of the electronics for data recordings and transmission via Bluetooth protocol.

For subsequent direct observation coding, video recordings were captured using a GoPro HERO8 Black camera (GoPro Inc., San Mateo, CA, USA), operating at a sampling frequency of 60 frames per second with a 1080p resolution and a wide field of view.

### Data collection

Participants were instructed to perform a series of daily activities, including sitting relaxed, sitting upright, standing, shuffling, walking slowly, walking at a normal pace, walking uphill, walking downhill, climbing stairs, descending stairs, walking with crutches and indoor cycling on a stationary bike. Each activity was to be performed twice within an overall recording duration of approximately 10 minutes. There were no restrictions on the duration or number of steps per activity, and no specific order was prescribed. Demonstrations of specific activities were provided only upon participant request. Otherwise, detailed instructions were intentionally avoided to encourage their individual natural execution of movements. Synchronisation between the insole sensors and video recordings was achieved using a fast heel drop, which served as the synchronisation event. To maintain data anonymisation, only the lower limbs were visible in the video recordings, ensuring that participants’ faces were not captured.

### Data processing

The Observer XT 16 software (Noldus Information Technology, Wageningen, Netherlands) was used to facilitate systematic direct observation coding of video recordings. Direct observation of video data is widely considered a gold standard method for physical activity classification [23–25], with the software enabling consistent, time-synchronised and reproducible annotation. A three-round annotation protocol was implemented to label participant’s physical activities. A complete list of the defined activity classes and activity types can be found in the S1 Table in the Supporting information file. In the first round, the synchronisation event (fast heel drop) and the start of each activity were encoded. In the second round, individual step and stair events were encoded. The third and final round focused on labelling straight and non-straight walking episodes, as well as a global quality check. Activities were encoded as state events (i.e., with a duration), while individual steps and stairs were marked as point events (i.e., without duration). Activities were coded as mutually exclusive and exhaustive. Hence, only one activity could be active at any given time, and the total duration of all activities summed to 100%. Consequently, the onset of a new activity automatically marked the end of the previous one. Steps were only counted if they were fully executed, including both a heel strike and toe-off. Straight walking episodes were identified as the WSS aims to analyse gait in standardised and comparable situations in real-world settings. A straight walking episode was defined as walking along a straight path without encountering obstacles (i.e., no visible change of direction or turn). In contrast, non-straight walking episodes involved changes in foot placement direction relative to the current walking trajectory. Shuffling was defined as movement in place without spatial displacement. Any step-like movements occurring during shuffling phases were not considered actual steps, as they did not contribute to a change in location.

The labelling task was divided between two independent investigators. To ensure consistency, both investigators annotated the first 23 videos, and inter-rater reliability was assessed by comparing their annotations. The mean Cohen’s Kappa was 0.88 (95% CI: 0.87–0.89), indicating high agreement.

Data processing for the WSS was performed onboard the digital interface using an embedded library (WalkinSense Analytics Library v1.0.2.2), which computes key spatiotemporal and kinematic gait characteristics (previously validated in [7,8]) as well as activity classifications. From the raw sensor inputs, the WSS extracted information on foot pose and orientation over time, together with global and regional pressure sums. A simple calibration procedure was performed using a 5-second static reference pose. This step was essential because the WSS is not inherently calibrated; it provides only relative variations with respect to a reference rather than absolute measurements. All data were therefore normalised to the reference pose and expressed as a percentage of the participants’ body weight, calculated from the combined pressure of both insoles.

**Fig 1:**
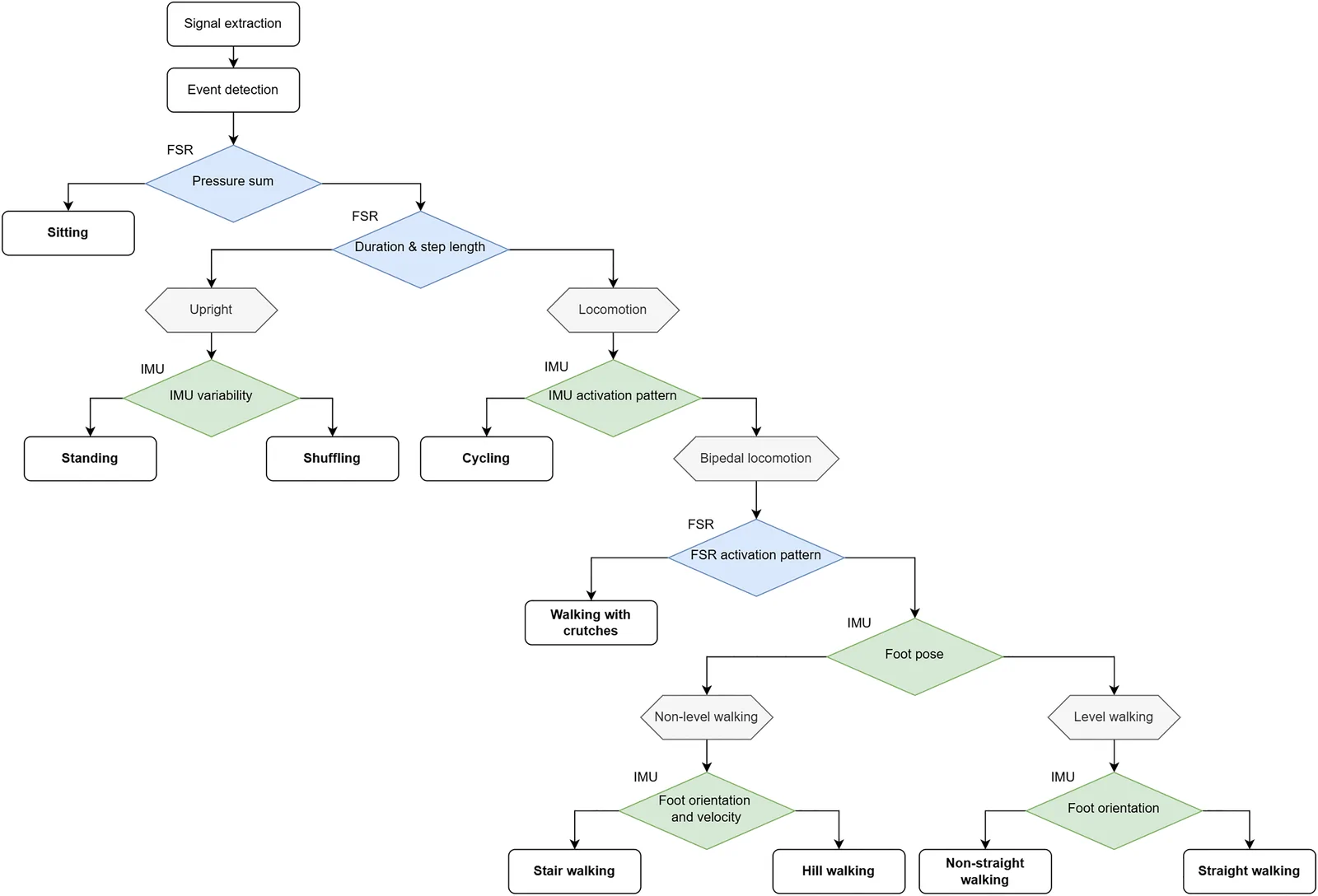
Classification tree for classifying daily activities using data from an instrumented insole. Abbreviations: FSR = force-sensitive resistor (insole); IMU = inertial measurement unit.

Subsequently, the data streams were analysed for classification into predefined activity classes. At the frame level, the system detected the presence of events by identifying substantial changes in the data streams (e.g., variations exceeding 50% of body weight in the pressure sum). In case of lack of change in the data stream, a maximum event duration criterion (e.g., 5 seconds) was applied, after which the segment was stored for further processing. For each detected event, features were extracted including, but not limited to, event duration (ms), average pressure (% body weight), positional and orientational variations (cm and degrees, respectively) and acceleration variation (m/s^2^). Based on these extracted features, events were classified into the predefined activity classes using a classification tree (Fig 1). The classification tree was developed from feature analyses of a representative subset of ten reference subjects from the study cohort.

The synchronisation event (fast heel drop) was automatically identified by detecting the maximum pressure-sum peak within the first 2-second time window.

In a final step, two labelled time series were exported for each system (WSS and ground truth): one capturing step and stair counts, and another detailing the observed activities. They were aligned by defining the start of the relative timestamp as the first observation following the synchronisation event. To match the time resolution of the WSS, the ground truth time series were therefore resampled to 100 Hz. In addition, minimum activity-duration thresholds were applied to the entire dataset to reduce the influence of very short, potentially ambiguous events. Results based on the 3-second minimum activity-duration threshold are presented in the main manuscript, whereas results for the 1-, 2-, 4-, and 5-second thresholds, as well as the unfiltered data (i.e., no minimum duration), are provided in the Supporting information file.

### Statistical analysis

Classifications from the WSS and ground truth were evaluated using both frame-by-frame and duration-based comparisons. Frame-by-frame agreement was quantified using overall accuracy and Cohen’s Kappa. Accuracy reflects the proportion of correctly classified frames, whereas Cohen’s Kappa quantifies agreement beyond chance and reduces the influence of class imbalance. Following common conventions, accuracy values above 80% and 90% can be interpreted as good and excellent, respectively, while Cohen’s Kappa values between 0.61 and 0.80 indicate substantial agreement and values between 0.81 and 1.00 indicate almost perfect agreement [26]. To assess frame-by-frame classification performance by activity class, precision, recall and F1-scores were calculated. These metrics were interpreted using established thresholds, with values ≥0.90 considered excellent, 0.80–0.89 good, 0.70–0.79 fair and <0.70 poor. Normalised confusion matrix heatmaps were generated to visualise the distribution of correctly and incorrectly classified frames by activity classes, with normalisation performed by the total count within each activity class to obtain relative frequencies. Moreover, for all episodes classified as walking by the WSS, we further assessed the system’s ability to differentiate between straight and non‑straight walking.

Duration-based agreement between the WSS and ground truth was evaluated using Mean Absolute Percentage Error (MAPE) scores for total activity class durations. MAPE scores provide an informative measure of individual-level agreement by accounting for each participant’s error while preventing cancellation of underestimation and overestimation [27]. MAPE values were interpreted as ≤10% highly accurate, 11–20% good, 21–50% reasonable and >50% inaccurate prediction [28]. Bland-Altman plots including a limit of agreement interval (LOA, ± 1.96 SD) were additionally used to assess agreement in total activity class durations between the two methods.

Finally, MAPE scores were calculated to compare step and stair counts between WSS and ground truth.

All processing steps and statistical analyses were performed in R (version 4.4.1) using RStudio (version 2024.09.1+394).

## Results

### Participant characteristics

Of the 104 participants tested, five were excluded from further analysis due to technical issues with the GoPro camera (*n* = 2) or with the insoles (*n* = 3), resulting in a final sample of 99 participants for the statistical analyses. The included participants were 51% female and had a mean ± *SD* age of 36 ± 12 years, a mean body mass of 71.7 ± 14.7 kg and a mean body height of 172 ± 10 cm.

### Frame-by-frame comparison

A mean ± *SD* overall accuracy of 0.87 ± 0.05 and a mean Cohen’s Kappa of 0.84 ± 0.07 were found, indicating an almost perfect level of agreement.

Table 1 summarises the classification performances by activity class. The results for the frame-by-frame comparisons indicate good performance for standing, level walking, hill walking and stair walking, and excellent performance for sitting, walking with crutches and indoor cycling.

**Table 1:** Classification performances by activity class.

Activity class	Frame-by-frame comparison			Duration-based comparison
	Precision	Recall	F1-score	MAPE scores (%)
Sitting	0.94	0.98	0.96	6.91
Standing	0.89	0.83	0.85	18.78
Level walking	0.83	0.94	0.88	15.76
Hill walking	0.92	0.71	0.80	23.84
Stair walking	0.97	0.72	0.83	24.36
Walking with crutches	0.93	0.91	0.93	6.79
Indoor cycling	0.97	0.90	0.93	8.48

**Fig 2:**
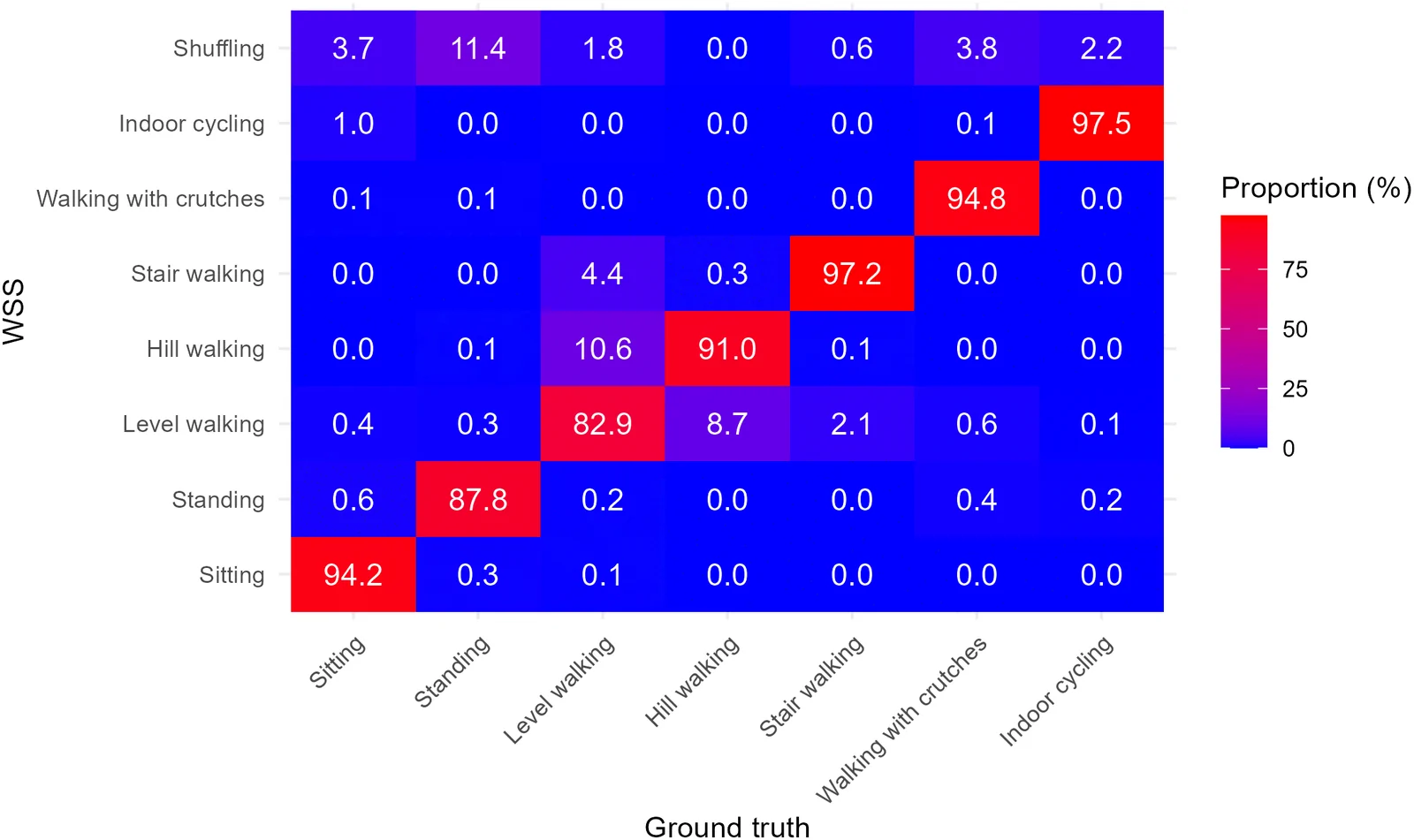
Normalised confusion matrix by activity class. Normalised by the total count within each activity class to obtain relative frequencies of correctly and incorrectly classified frames by activity class. Abbreviation: WSS = WalkinSense system.

Fig 2 shows the distribution of correctly and incorrectly classified frames by activity class. Overall, high relative frequencies (generally above 80%) along the main diagonal indicate good frame-by-frame classification performance across all activity classes. Indoor cycling and stair walking were predominantly classified correctly, with minimal confusion. Misclassifications occurred mainly between level walking, hill walking and stair walking. Besides, sitting, standing, walking with crutches and indoor cycling showed some misclassification with shuffling.

When splitting level walking into straight and non-straight walking, the WSS showed strong performance in identifying straight walking episodes, with precision, recall, and F1‑scores of 0.82, 0.96 and 0.88, respectively.

### Duration-based comparison

Based on the total durations of each activity class (mean total durations presented in S2 Table in the supporting information file), the MAPE scores indicate highly accurate prediction accuracy (≤ 10%) for sitting, walking with crutches and indoor cycling (Table 1). Standing and level walking show good prediction accuracy (11–20%), while hill walking and stair walking demonstrate only a reasonable prediction performance (21–50%).

**Fig 3:**
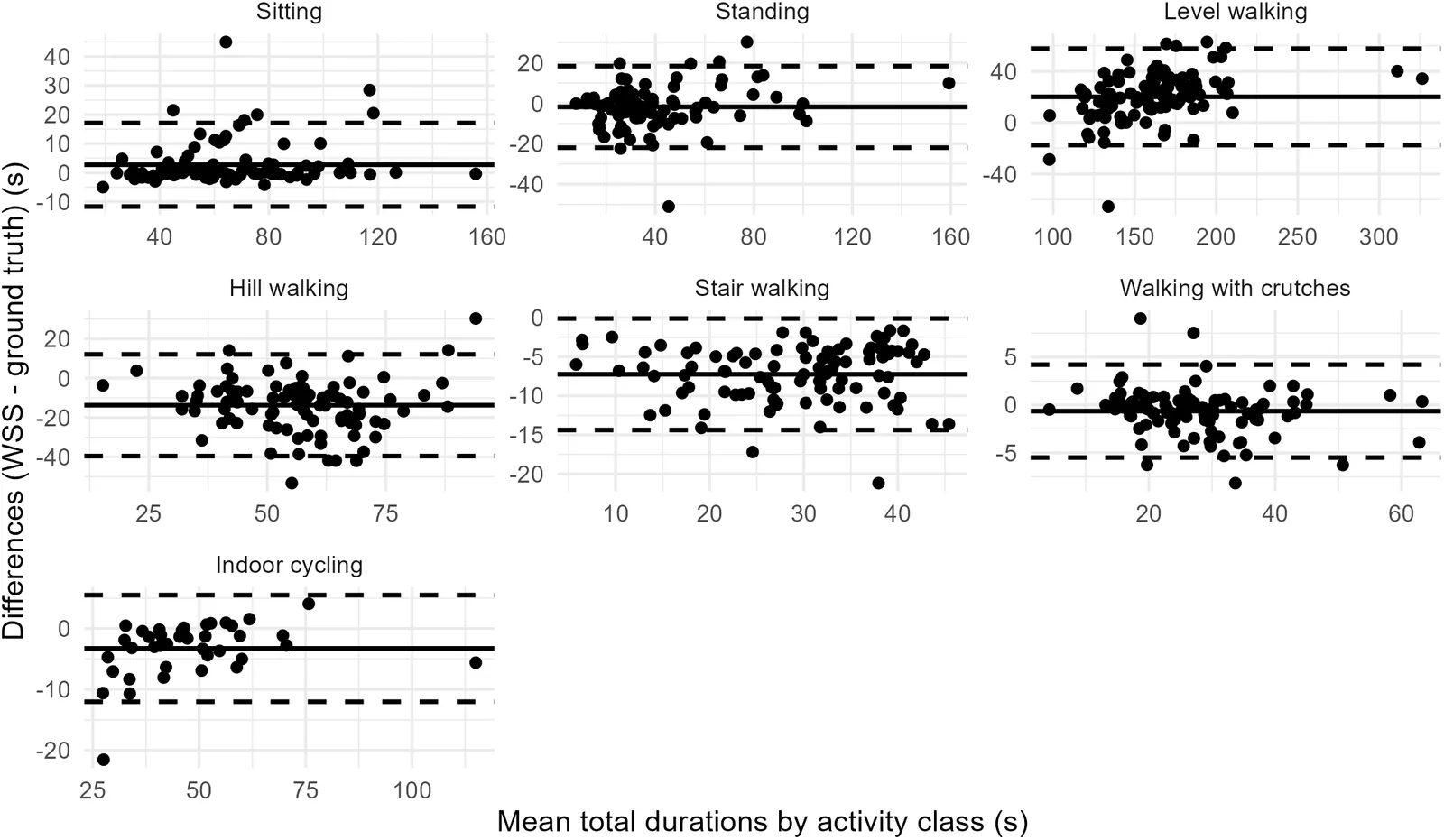
Bland-Altman plots for total duration comparisons by activity class. The solid line represents the mean difference, while the dashed lines represent the upper and lower limits of agreement (±1.96 *SD*). Abbreviation: WSS = WalkinSense system.

The Bland-Altman plots in Fig 3 illustrate good agreement between the WSS and the ground truth for walking with crutches (mean difference = −0.7 s; LOA = −5.5 to 4.2 s), standing (−1.8 s; −21.9 to 18.4 s), indoor cycling (−3.3 s; −12.0 to 5.5 s) and sitting (2.7 s; −11.7 to 17.1 s). In contrast, level walking showed a systematic overestimation (20.1 s; −17.5 to 57.7 s), whereas hill walking (−13.7 s; −39.6 to 12.1 s) and stair walking (−7.3 s; −14.4 to −0.1 s) were generally underestimated.

### Step and stair count comparison

Mean ± *SD* total step counts were 544 ± 75 steps for the ground truth and 545 ± 77 steps for the WSS, while mean ± *SD* stair counts were 85 ± 7 stairs for the ground truth and 63 ± 11 stairs for the WSS. The corresponding MAPE scores were 2.36% for total step count, indicating an excellent prediction, and 26.05% for stair count, indicating only a reasonable prediction accuracy.

## Discussion and conclusion

The present study evaluated the accuracy of a wireless instrumented insole that combines plantar pressure and inertial data to classify ADLs in a semi free-living setting. Overall, the system achieved near‑perfect agreement at the whole‑dataset level, underscoring its potential for real-world monitoring of daily activities.

Activities with distinctive signal patterns, such as sitting, indoor cycling, and walking with crutches, were identified with very high accuracy in both frame‑by‑frame and duration‑based analyses. Level walking was also well recognised. However, differentiating level from non‑level walking (hill and stairs) was more challenging, leading to some confusions between these classes. Consequently, hill and stair walking exhibited the lowest recall (sensitivity), indicating systematic under‑detection of these activities, whereas level walking showed the lowest precision, suggesting that it was most frequently confused with other walking-related activities. This pattern is confirmed by the confusion matrix, which shows that level walking was most frequently misclassified as hill walking and vice versa. Nevertheless, all these confusions occurred within the broader category of walking, meaning that when the focus is on identifying walking behaviour, irrespective of the slope, the classifier achieved consistently high accuracy across all walking‑related activities. Standing performance was good but not excellent, mainly due to confusion with shuffling. In real-life, and likewise in our semi free-living setting, participants do not remain completely motionless, and such subtle postural adjustments were sometimes difficult to distinguish in the video-based ground truth as well. Moreover, shuffling behaviour occurred frequently outside the instructed activities, particularly during short transition phases (e.g., getting on or off the bike, picking up crutches, repositioning before walking or sitting), further increasing overlap between shuffling and other activity classes such as walking with crutches, indoor cycling and sitting.

Moreover, the system’s ability to differentiate between straight and non‑straight walking was assessed for all episodes that the WSS classified as walking. The system showed strong performance in identifying straight walking episodes, indicating that such standardised and comparable situations can be reliably captured in real-world settings. These episodes provide suitable conditions for analysing specific gait parameters that are typically used for clinical or rehabilitation monitoring.

Despite a clear underestimation of stair counts, step detection itself was robust. Many stair steps were correctly detected as steps but were not recognised in a stair‑walking context. Instead, they were assigned to other walking activities (e.g., level or hill walking). Thus, misclassifications affected stair count and activity classification rather than overall step counts, which remained accurate.

The comparison of different minimum activity‑duration thresholds revealed that overall classification performance improved as the threshold increased. Both overall accuracy and Cohen’s Kappa rose steadily, peaking at 0.90 and 0.88 for a 5‑second threshold (S3 Table in the supporting information file). Frame‑by‑frame precision, recall, and F1‑scores generally improved with longer thresholds (S4 Table in the Supporting information file), with the sole exception of stair‑walking recall, which declined slightly at 5 seconds. This likely reflects the environmental constraints of our protocol: on staircases with intermediate landings, many real stair bouts were short-duration activities. Thus, applying a longer threshold removed these correctly classified but short episodes, lowering recall for stair walking. For the other activities, longer thresholds filtered out short, ambiguous segments, reducing misclassifications. Consistently, MAPE scores decreased with higher thresholds (S5 Table in the Supporting information file), indicating more accurate duration estimation and confirming the stabilising effect of applying a minimum activity-duration threshold.

Our overall accuracy is consistent with the two studies that have incorporated a semi free‑living component, where the challenges of natural behavioural variability and environmental noise typically reduce performance relative to controlled laboratory settings. Hegde et al. [2], for example, analysed a full day of free‑living data from 15 participants using an insole equipped with force‑sensitive resistors and an accelerometer. While their multinomial logistic discrimination model achieved 97% accuracy in the laboratory, accuracy dropped to 83% in free‑living conditions. Importantly, classification was limited to sedentary, standing and stepping activities due to the constraints of the ActivPAL reference device, which is furthermore less accurate than direct observation. Similarly, Moufawad El Achkar et al. [20] evaluated a pressure‑inertial insole system during 4 hours of home‑based free‑living in 10 participants and achieved 93% accuracy using a threshold‑based model, again restricted to only three activity categories (sitting/lying, standing and walking). Their comparison device (Physilog) provides sensitivity and specificity above 90% [20] but still below the standard of direct observation. Notably, the same model achieved 97% when tested in a controlled environment [29], illustrating how behavioural variability and environmental noise naturally reduce performance when transitioning from structured laboratory conditions to ecologically valid settings.

Overall accuracies from further studies employing ML and DL models, typically based on small, highly controlled datasets, ranged from 75 to 100% for ML approaches [16,17] and 92 to 99.6% for DL approaches [1,16], respectively. These values, however, must be interpreted in the context of markedly different study designs. They largely reflect idealised laboratory conditions with ≤34 participants performing limited activities. In contrast, our study involved 99 participants completing a wide range of ADLs across indoor and outdoor environments in a semi free‑living protocol, introducing natural variability in movement execution, pace, environmental conditions and incidental movements, factors that create a more realistic but inherently more challenging classification problem. Methodological differences such as calibration procedures, barometric sensing, IMU placement, sampling rates and processing pipelines (e.g., segmentation, feature extraction and filtering strategies) likely further contribute to discrepancies in accuracy. Crucially, the superior performance of complex ML and DL models in prior work does not necessarily translate to large, heterogeneous, noisy datasets, where such models may overfit and provide limited interpretability. Given our directly observed, ecologically valid dataset, we made a deliberate methodological choice to use a classical classification tree approach, prioritising robustness, transparency and translational feasibility over architectural complexity and providing a more realistic benchmark of achievable performance under everyday rehabilitation‑relevant conditions.

A key strength of the WalkinSense system lies in its integrated use of pressure and inertial data, enabling the classification model to capitalise on complementary sensor information. Pressure patterns support reliable discrimination between body postures such as sitting and standing, while inertial signals are particularly effective for identifying dynamic activities, including distinguishing level from non‑level walking. Combining these streams enables more nuanced movement characterisation and enhances robustness, consistent with prior evidence demonstrating that pressure-inertial combinations enhances classification performance [11]. Importantly, the present study evaluates this multimodal system under ecologically valid conditions, using a large sample (*n* = 99) performing a broad set of ADLs across indoor and outdoor environments in a semi free‑living protocol. This design strengthens the generalisability of the findings and situates our results as a realistic benchmark for the performance of smart‑insole systems under conditions approximating everyday rehabilitation and mobility contexts. Limitations include a study population that consisted exclusively of healthy adults and classification performance may differ in populations with gait‑altering conditions. Second, although the protocol aimed to approximate natural behaviour, the semi free‑living setting remains more structured than genuine real‑world conditions and performance may therefore be lower in fully unconstrained daily environments. Finally, although the insole‑based design reduces participant burden by integrating the sensors into regular footwear, it also imposes a limitation, as shoes are often not worn at home, resulting in missing data during these periods.

Building on the current rule‑based and classical modelling approach, adopting supervised ML methods (e.g., SVM, k‑NN, decision trees and random forests) is a logical next step, as these models have already demonstrated strong performance for insole‑based classification in small, controlled datasets [30]. Importantly, the present study provides the first large, heterogeneous, directly observed dataset under ecologically valid conditions, establishing a necessary methodological benchmark before transitioning to more complex architectures. With this foundation now in place, future work can systematically evaluate whether higher‑capacity models can maintain reliability and generalisability when confronted with the behavioural variability and noise characteristic of semi free‑living environments. In particular, SVMs offer several practical advantages over DL architectures, including faster training times, reduced dependence on very large datasets and clearly defined decision boundaries, making them suitable for daily‑activity classification in real-world settings [16]. In parallel, future research should (i) refine features for non‑level walking, (ii) optimise IMU placement and calibration routines to strengthen signal quality and (iii) validate performance in fully free‑living settings to assess model stability and responsiveness to behavioural variability. Together, these efforts will advance the system’s real‑world applicability and strengthen performance for slope‑ and stair‑related activities, while enabling a controlled and scientifically grounded progression toward more complex modelling strategies.

## Supporting information

S1 Table List of daily activity classes and types. (DOCX)

S2 Table Mean total durations by activity class and measurement device. (DOCX)

S3 Table Frame-by-frame classification agreement, applying different minimum activity-duration thresholds. (DOCX)

S4 Table Frame-by-frame classification statistics by activity class, applying different minimum activity-duration thresholds. (DOCX)

S5 Table MAPE scores for total duration comparisons by activity class, applying different minimum activity-duration thresholds. (DOCX)

S1 Fig Normalised confusion matrices by activity class, applying different minimum activity-duration thresholds. (DOCX)

S2 Fig Bland-Altman plots for total duration comparisons by activity class, applying different minimum activity-duration thresholds. (DOCX)
