# Supplementary material for: Classification of daily activities using a wireless instrumented insole (WalkinSense) in a semi free-living setting
Source: PLoS One. 2026 Sep 16;21(9):e0357961. doi: 10.1371/journal.pone.0357961 (PMC13580994; doi:10.1371/journal.pone.0357961)
Supplement: Supplementary file 1 [file pone.0357961.s001.docx]

**S1 Table. List of daily activity classes and types.**

| **Activity class** | **Activity type** |
| --- | --- |
| Sitting | Sitting relaxed |
|  | Sitting upright |
| Standing | Standing |
| Shuffling | Shuffling |
| Level walking | Walking slowly straight |
|  | Walking straight |
|  | Walking slowly non-straight |
|  | Walking non-straight |
| Hill walking | Walking uphill |
|  | Walking downhill |
| Stair walking | Climbing stairs |
|  | Descending stairs |
| Walking with crutches | Walking with crutches |
| Indoor cycling | Indoor cycling |
