# Supplementary material for: Classification of daily activities using a wireless instrumented insole (WalkinSense) in a semi free-living setting
Source: PLoS One. 2026 Sep 16;21(9):e0357961. doi: 10.1371/journal.pone.0357961 (PMC13580994; doi:10.1371/journal.pone.0357961)
Supplement: Supplementary file 2 [file pone.0357961.s002.docx]

**S2 Table. Mean total durations by activity class and measurement device.**

| **Activity class** | **Ground truth (seconds)**  *Mean (SD)* | **WSS (seconds)**  *Mean (SD)* |
| --- | --- | --- |
| Sitting | 63 (25) | 66 (26) |
| Standing | 40 (23) | 39 (25) |
| Level walking | 151 (32) | 172 (39) |
| Hill walking | 63 (16) | 50 (15) |
| Stair walking | 32 (10) | 25 (10) |
| Walking with crutches | 28 (11) | 27 (11) |
| Indoor cycling | 50 (16) | 46 (17) |
