# Supplementary material for: Classification of daily activities using a wireless instrumented insole (WalkinSense) in a semi free-living setting
Source: PLoS One. 2026 Sep 16;21(9):e0357961. doi: 10.1371/journal.pone.0357961 (PMC13580994; doi:10.1371/journal.pone.0357961)
Supplement: Supplementary file 3 [file pone.0357961.s003.docx]

**S3 Table. Frame-by-frame classification agreement, applying different minimum activity-duration thresholds.**

| **Minimum activity-duration threshold** | **Accuracy**  *Mean (SD)* | **Cohen’s Kappa**  *Mean (SD)* |
| --- | --- | --- |
| None | 0.81 (0.05) | 0.76 (0.07) |
| 1s | 0.82 (0.05) | 0.77 (0.07) |
| 2s | 0.85 (0.05) | 0.81 (0.07) |
| 3s | 0.87 (0.05) | 0.84 (0.07) |
| 4s | 0.89 (0.05) | 0.86 (0.07) |
| 5s | 0.90 (0.05) | 0.88 (0.07) |
