# Supplementary material for: Classification of daily activities using a wireless instrumented insole (WalkinSense) in a semi free-living setting
Source: PLoS One. 2026 Sep 16;21(9):e0357961. doi: 10.1371/journal.pone.0357961 (PMC13580994; doi:10.1371/journal.pone.0357961)
Supplement: Supplementary file 4 [file pone.0357961.s004.docx]

**S4 Table.** **Frame-by-frame classification statistics by activity class, applying different minimum activity-duration thresholds.**

| **Activity class** | **Minimum activity-duration threshold** | | | | | |
| --- | --- | --- | --- | --- | --- | --- |
|  | **None** | **1s** | **2s** | **3s** | **4s** | **5s** |
|  | **Precision** | | | | | |
| Sitting | 0.93 | 0.93 | 0.94 | 0.94 | 0.95 | 0.96 |
| Standing | 0.82 | 0.82 | 0.84 | 0.89 | 0.91 | 0.92 |
| Level walking | 0.77 | 0.78 | 0.81 | 0.83 | 0.86 | 0.88 |
| Hill walking | 0.84 | 0.85 | 0.90 | 0.92 | 0.93 | 0.94 |
| Stair walking | 0.93 | 0.93 | 0.94 | 0.97 | 0.98 | 0.99 |
| Walking with crutches | 0.92 | 0.93 | 0.93 | 0.93 | 0.95 | 0.96 |
| Indoor cycling | 0.90 | 0.97 | 0.97 | 0.97 | 0.97 | 0.98 |
|  | **Recall** | | | | | |
| Sitting | 0.96 | 0.96 | 0.97 | 0.98 | 0.98 | 0.99 |
| Standing | 0.74 | 0.76 | 0.81 | 0.83 | 0.85 | 0.86 |
| Level walking | 0.88 | 0.89 | 0.92 | 0.94 | 0.95 | 0.95 |
| Hill walking | 0.67 | 0.67 | 0.69 | 0.71 | 0.73 | 0.74 |
| Stair walking | 0.67 | 0.68 | 0.71 | 0.72 | 0.77 | 0.63 |
| Walking with crutches | 0.88 | 0.88 | 0.90 | 0.91 | 0.93 | 0.94 |
| Indoor cycling | 0.88 | 0.88 | 0.89 | 0.90 | 0.91 | 0.92 |
|  | **F1-score** | | | | | |
| Sitting | 0.94 | 0.94 | 0.95 | 0.96 | 0.96 | 0.97 |
| Standing | 0.77 | 0.78 | 0.81 | 0.85 | 0.87 | 0.88 |
| Level walking | 0.82 | 0.83 | 0.86 | 0.88 | 0.90 | 0.91 |
| Hill walking | 0.73 | 0.74 | 0.77 | 0.80 | 0.81 | 0.82 |
| Stair walking | 0.77 | 0.78 | 0.81 | 0.83 | 0.87 | 0.91 |
| Walking with crutches | 0.90 | 0.90 | 0.92 | 0.93 | 0.94 | 0.95 |
| Indoor cycling | 0.92 | 0.92 | 0.92 | 0.93 | 0.94 | 0.94 |
