# Supplementary material for: Classification of daily activities using a wireless instrumented insole (WalkinSense) in a semi free-living setting
Source: PLoS One. 2026 Sep 16;21(9):e0357961. doi: 10.1371/journal.pone.0357961 (PMC13580994; doi:10.1371/journal.pone.0357961)
Supplement: Supplementary file 5 [file pone.0357961.s005.docx]

**S5 Table.** **MAPE scores for total duration comparisons by activity class, applying different minimum activity-duration thresholds.**

| **Activity class** | **Minimum activity-duration threshold** | | | | | |
| --- | --- | --- | --- | --- | --- | --- |
|  | **None** | **1s** | **2s** | **3s** | **4s** | **5s** |
| Sitting | 7.66 | 7.69 | 7.35 | 6.91 | 5.92 | 5.37 |
| Standing | 20.86 | 21.09 | 20.57 | 18.78 | 17.38 | 15.99 |
| Level walking | 16.23 | 16.45 | 15.99 | 15.76 | 13.13 | 11.50 |
| Hill walking | 23.23 | 23.17 | 24.38 | 23.84 | 22.35 | 22.57 |
| Stair walking | 27.49 | 27.09 | 24.71 | 24.36 | 19.68 | 14.57 |
| Walking with crutches | 8.12 | 8.06 | 7.31 | 6.79 | 6.89 | 6.32 |
| Indoor cycling | 9.73 | 9.72 | 9.15 | 8.48 | 7.51 | 6.61 |
