# Supplementary material for: Classification of daily activities using a wireless instrumented insole (WalkinSense) in a semi free-living setting
Source: PLoS One. 2026 Sep 16;21(9):e0357961. doi: 10.1371/journal.pone.0357961 (PMC13580994; doi:10.1371/journal.pone.0357961)
Supplement: Supplementary file 6 [file pone.0357961.s006.docx]

**S1 Fig. Normalised confusion matrices by activity class, applying different minimum activity-duration thresholds.**
